# Effect and Mechanism of Dragon's Blood on Wound Healing of Patients with Stress Hand Injury

**DOI:** 10.1155/2023/6122331

**Published:** 2023-01-21

**Authors:** Shulin Sun, Peng Wang, Kaifeng Yue, Qipeng Yi, Xinjun Xie, Xianmin Xie

**Affiliations:** ^1^Hunan University of Chinese Medicine, Changsha 410208, Hunan, China; ^2^Department of External Hand Trauma, The First Affiliated Hospital of Hunan University of Chinese Medicine, No. 95, Shaoshan Middle Road, Yuhua District, Changsha City, Hunan Province, China

## Abstract

This study aimed to explore the effect and mechanism of Dragon's Blood on wound healing in patients with a pressure hand injury. A total of 120 patients with pressure hand injury treated in our hospital were randomly divided into two groups. Sixty patients in the control group were dressed with sterile gauze, and 60 patients in the observation group were smeared with blood exhaustion. The clinical effects and serological indexes of the two groups were compared, and the mechanism of wound healing was analyzed. The results showed that the treatment effective rate of the control group was 80% and that of the observation group was 93.33%. The treatment effective rate of the observation group was dramatically higher (*P* < 0.05). The number of patients with good granulation tissue in the observation group was 53, which was dramatically greater than that in the control group. The number of patients with a small amount of wound exudation was 51, which was dramatically greater than that in the control group (*P* < 0.05). After treatment, the levels of matrix metalloproteinase (MMP-3), vascular endothelial growth factor (VEGF), and transforming growth factor B1 (TGF-B1) in the observation group increased more dramatically (*P* < 0.05). The level of tissue inhibitor of metalloproteinase-1 (TIMP-1) decreased to 617.23 ng/L in the observation group, and the degree of reduction was more obvious (*P* < 0.05). Notably, Dragon's Blood promoted wound healing at the injury site by increasing the levels of MMP-3, VEGF, and TGF-B1and decreasing TIMP-1. The area of wound reduction in the observation group was 0.27 cm^2^, and the reduction was more obvious (*P* < 0.05). The healing time of pressure hand injury in the observation group was 15.27 days, which was dramatically shorter (*P* < 0.05). In summary, Dragon's Blood had a good effect on the healing of the injured site in patients with pressure hand injury, which is worthy of promotion.

## 1. Introduction

Hands are the main organs of labor and work, and most work crush injuries occur in both hands or arms [1, 2]. Hand injury will seriously affect people's daily life and social work, bringing physical pain and economic burden to patients [3, 4]. The pressure injury of the hand has a great impact on the hand, and it takes a long time to recover the wound, which is prone to form chronic refractory wounds so that the wound cannot be properly repaired. The recovery is not timely, and it is difficult to achieve a normal functional state. The inflammatory reaction persists and does not heal, which seriously bothers patients [5–7]. If the wound treatment of pressure injury is not timely, it can easily become a chronic refractory wound, which seriously affects wound recovery [8, 9].

The formation mechanism of chronic refractory wounds is complex, and inflammatory reactions and enzymes affect the recovery of wounds [10]. On the one hand, the persistence of a state of excessive inflammatory response can cause the wound to continue to heal. Granulation tissue is difficult to form for a long time, and the self-repair function of the body is affected, which means it cannot meet the conditions needed for wound recovery. On the other hand, stress wounds are prone to ischemia and hypoxia, and matrix metalloproteinases are constantly activated, which will cause the decomposition of cell growth factors and affect the growth and proliferation of epidermal cells, making wound recovery difficult [11]. Western medicine usually adopts debridement treatment, surgical intervention, electrical stimulation, biotechnology, and negative pressure wound therapy to carry out wound recovery treatment [12]. Western medicine treatment causes great damage to the body, with higher costs and more side effects. Compared with Western medicine, traditional Chinese medicine treatment has less damage, fewer side effects, and significant effects and has been widely recognized by patients [13]. Dragon's Blood (DB) is a traditional Chinese medicine (TCM) herb used to treat pressure wounds that can promote blood circulation and dispersing stasis, generate muscle, and collect ulcers and has a good therapeutic effect on all stages of pressure ulcers [14]. DB can promote blood circulation at the wound site, improve immune cells, promote the formation of fibroblasts by promoting the expression of VEGF, and improve the epidermal repair ability near the wound. Wound repair involves the inflammatory response, tissue repair, scar formation, and other processes, and a variety of cytokines are involved in wound repair and can promote wound repair. Wet application of DB on pressure wounds can reduce the probability of infection, promote the growth of granulation tissue, clean the wound, achieve decomposition and muscle building, and promote wound recovery [15].

In this study, the effect of Dragon's Blood on wound healing in patients with pressure hand injury was discussed. The innovation of this study was that it explored the mechanism of wound recovery of pressure hand injury treated with sterile gauze and Dragon's Blood based on the serological indicators of the two groups of patients. This study aimed to provide clinical guidance for wound recovery treatment in patients with a pressure hand injury and to provide data support for theoretical research on wound healing in patients with a pressure hand injury.

## 2. Materials and Methods

### 2.1. The Research Object

The subjects of this study were 120 patients with pressure hand injuries treated in our hospital from September 2020 to January 2022. They were divided into two groups by the random number method, including 60 patients in the control group, whose wounds were dressed with sterile gauze. The male-to-female ratio of the control group was 34/26, with an average age of 31.33 ± 6.75 years, and the length of education was 12.33 ± 2.65 years. There were 60 patients in the observation group. The wounds were smeared with Dragon's Blood, and the male-to-female ratio of the subjects in the observation group was 33/27. The average age was 31.46 ± 6.76 years. The length of education was 12.54 ± 2.63 years, and there was no significant difference in general information between the two groups (*P* < 0.05). The therapeutic effect and wound recovery were compared among the subjects included in the study. This study was approved by the Ethics Committee of our hospital.

Inclusion criteria: (i) patient who had complete inpatient treatment data; (ii) patients over 18 years old; (iii) patient's hand suffered from pressure injury; (iv) patient who had no hereditary diseases; (v) patient who had no immune disease; (vi) patient who had no communication disorder; (vii) patients and their family members signed informed consent.

Exclusion criteria: (i) patients' medical records and follow-up data were not complete; (ii) patients with vital organ diseases; (iii) patients with other nonpressure hand injuries; (iv) patients who had coagulation dysfunction; (v) pregnant or lactating women; (vi) patients who had an inherited disease; (vii) patients who were allergic to Dragon's Blood; (viii) patients who were unwilling to participate in this study.

### 2.2. Methods of Dressing Treatments

All patients in the control group received routine anti-infection treatment after debridement. From the first day after debridement, sterile gauze (Shanghai Bejin Yangheng Products Co., LTD.) was changed every morning and evening. Sodium chloride (0.9%) cotton balls were used to clean the wound, and iodophor cotton balls were used to disinfect the wound after debridement. After disinfection, the wound was covered and bandaged with sterile gauze and fixed with adhesive tape for 30 min each time twice a day.

In the observation group, debridement was performed on the first day after surgery. Dragon's Blood dressing was changed every morning and evening. A 0.9% sodium chloride cotton ball was used to clean the wound after the debridement operation, and an iodophor cotton ball was used to disinfect the wound. After disinfection, the wound was exposed, and a Dragon's Blood capsule (Yunnan Yunhe Pharmaceutical Co., LTD., Sinopharm approval number Z53020999, 0.3 g/capsule) was opened and evenly spread on sterile yarn. Sterile gauze was laid flat on the wound surface after debridement. The wound was covered with sterile gauze and fixed with adhesive tape for 30 min each time, twice a day.

### 2.3. Observation Indicators

General data, including sex, age, and years of education, of patients in different groups, were compared.

The clinical efficacy was compared, including cure, remarkable effect, effective, and invalid. Cure referred to that all local symptoms disappeared, a remarkable effect referred to that the local symptoms were dramatically alleviated, an effective referred to that the local symptoms were reduced, and an invalid referred to that the local symptoms showed no obvious change or symptom aggravation. The treatment effective rate (ER) was calculated. The ER was calculated as shown in (1), where *Recovery* refers to the number of cured patients, *Remarkable effect* refers to the number of remarkable effects, *Effective* refers to the number of effective patients, and *Total* refers to the total number of patients.

(1) $$\begin{array}{c} \text{Efficient}=\frac{\text{Recovery}+\text{Remarkable effect}+\text{Effective}}{\text{Total}}\times 100\%. \end{array}$$

The wound recovery of the two groups was compared, mainly including granulation tissue, wound exudation amount, and wound edema. The granulation tissue conditions included good granulation, fresh granulation, and dull granulation. It was graded as a small amount of wound seepage without permeating one gauze, a medium amount of permeating one gauze, or a large amount of permeating two or more gauze. Wound edema included no edema, degree of edema<1 cm, and degree of edema >1 cm.

The serological indexes of the two groups were compared, mainly including MMP-3, T1MP-1, VEGF, and TGF-B1. Five milliliters of fasting peripheral venous blood were collected from the patient and centrifuged in a Danish Labogene desktop mini centrifuge. The centrifugation time and speed were set to 15 min and 5,000 R per minute, respectively. Then, the supernatant after centrifugation was collected for detection of serum levels of MMP-3, T1MP-1, VEGF, and TGF-B1. The detection of the above categories of indicators was carried out by several senior inspectors in strict accordance with the relevant instructions, and the enzyme-linked immunosorbent assay was adopted to detect the serological indicators. Then, the serum levels of MMP-3, T1MP-1, VEGF, and TGF-B1 were statistically compared between the two groups. The wound area and wound healing time of patients were compared.

### 2.4. Method of Statistics

Excel 2016 was used to record and summarize the data. SPSS 20.0 was used for data statistics and analysis. The mean ± standard deviation (*X* ± *S*) represents the measurement data, and a *t*-test was used. Percentage (%) is the representation of count data using the chi-square test. *P* < 0.05 was considered statistically significant.

## 3. Results

### 3.1. Comparison of General Data between the Two Groups

Table 1 shows the comparison of general data between the two groups. The mean systolic blood pressure of the control group was 123.24 ± 12.79 mmHg, and the mean systolic blood pressure of the observation group was 124.66 ± 12.38 mmHg. The diastolic blood pressure of the control group was 82.33 ± 7.79 mmHg and that of the observation group was 81.46 ± 7.56 mmHg. The blood glucose of the control group was 4.33 ± 1.69 mmol/L and that of the observation group was 4.54 ± 1.65 mmol/L. General information was not significantly different between the two groups (*P* > 0.05) and was comparable.

### 3.2. Stage of Pressure Injury

**Figure 1:**
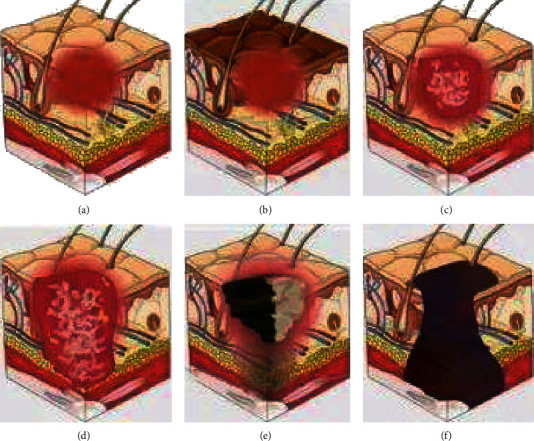
Staging diagram of pressure injury: (a) is stage 1, (b) is stage 2, (c) is stage 3, (d) is stage 4, (e) is stage 5, and (f) is stage 6.

Figure 1 shows the staging of pressure injury. There are six stages of pressure injury. The first stage was stress injury, mainly manifested as skin redness, swelling, heat, and pain, without any deep tissue damage. In the second stage, local skin tissue exhibited induration, mass, blisters, and no musculoskeletal destruction. The third stage was superficial tissue ulceration, which was characterized by local skin defects and abscess formation of skin and subcutaneous tissue pus. The fourth stage was a deep ulcer, not only skin, subcutaneous tissue necrosis, and ulcer but also necrosis of skeletal muscle destruction. Stage five was deep damage. Stage 6 was defined as a deep tissue injury.

### 3.3. Comparison of Clinical Efficacy between the Two Groups

**Figure 2:**
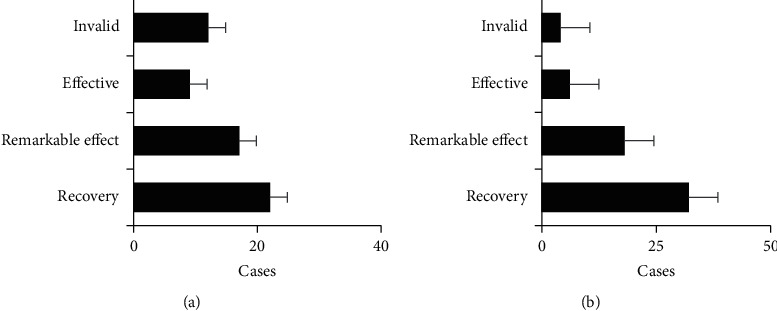
Comparison of clinical efficacy between the two groups: (a) is the control group; (b) is the observation group.

**Figure 3:**
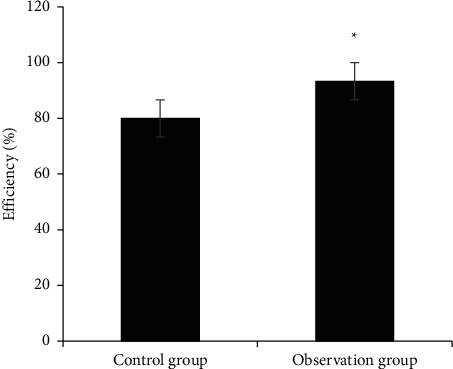
Comparison of treatment response rates between the two groups (^*∗*^ denotes that compared with control patients, *P* < 0.05).

Figure 2 shows the comparison of clinical efficacy between the two groups, and Figure 3 shows the comparison of treatment effective rate between the two groups. In the control group, 22 cases were recovered, 17 cases were effective, 9 cases were effective, and 12 cases were ineffective. In the observation group, 32 cases were recovered, 18 cases were effective, 6 cases were effective, and 4 cases were ineffective. The number of recovered patients in the observation group was dramatically higher than that in the control group, and the number of ineffective patients was dramatically lower than that in the control group (*P* < 0.05). The ERs of groups A and B were 80% and 93.33%, respectively, suggesting statistically observable differences (*P* < 0.05).

### 3.4. Comparison of Wound Recovery

**Figure 4:**
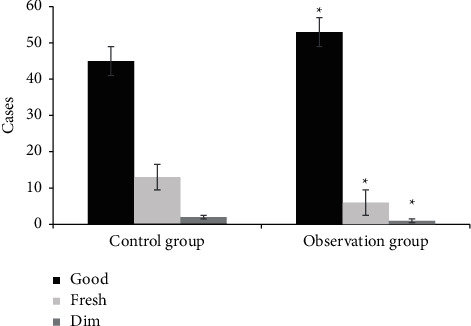
Comparison of wound granulation tissue between the two groups (^*∗*^ denotes that compared with control patients, *P* < 0.05).

**Figure 5:**
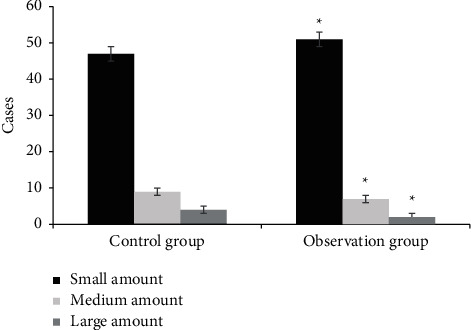
Comparison of wound exudation volume between the two groups (^*∗*^ denotes that compared with control patients, *P* < 0.05).

**Figure 6:**
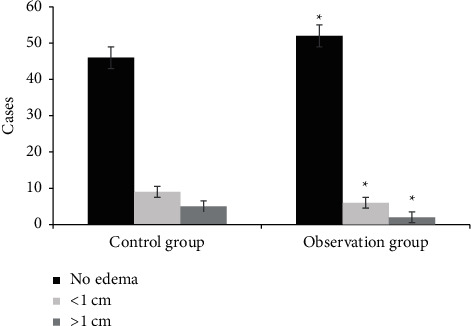
Comparison of wound edema degree between the two groups (^*∗*^ denotes that compared with control patients, *P* < 0.05).

Figure 4 shows a comparison of wound granulation tissues. In group A, 45 patients had good granulation tissue, 13 patients had fresh granulation tissue, and 2 patients had dull granulation tissue after treatment. After treatment, 53 patients in group B had good granulation tissue, 6 patients had fresh granulation tissue, and 1 patient had dull granulation tissue. The number of patients with good granulation tissue in group B was much higher (*P* < 0.05).  Figure 5 shows the comparison of wound exudation volume. In group A, after treatment, there were 47 cases with a small amount of wound exudation, 9 cases with a medium amount of wound exudation, and 4 cases with a large amount of wound exudation. In group B, there were 51 cases with a small amount of wound exudation, 7 cases with a medium amount of wound exudation, and 2 cases with a large amount of wound exudation. The number of patients with a small amount of wound exudation was dramatically higher than that of group A (*P* < 0.05).  Figure 6 shows the comparison of the degree of wound edema. After treatment, 46 patients in group A had no edema. There were 9 patients with an edema degree of <1 cm and 5 patients with an edema degree of >1 cm. After treatment, 52 patients in group B had no edema. There were 6 patients with an edema degree of <1 cm and 2 patients with an edema degree of >1 cm. The number of patients without edema in group B was higher, which was obviously observed (*P* < 0.05).

### 3.5. Serological Indexes of the Two Groups

**Figure 7:**
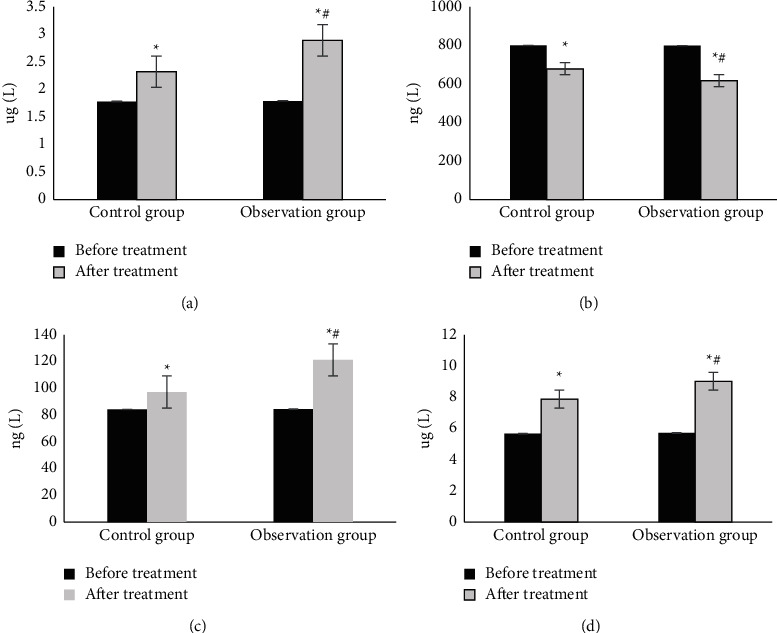
Comparison of serological indicators. (a) MMP-3, (b) T1MP-1, (c) VEGF, and (d) TGF-B1; ^*∗*^ and ^#^ indicate *P* < 0.05 compared to the value before treatment and the value in group (a), respectively.

As demonstrated in Figure 7, serological indicators of patients were compared. No significant difference was found before the treatment in the levels of MMP-3, T1MP-1, VEGF, and TGF-B1 (*P* > 0.05). The pretreatment levels of MMP-3, VEGF, and TGF-*β*1 were dramatically increased for all patients in the different groups, but this phenomenon in group B was more visible (*P* < 0.05). After treatment, the T1MP-1 level decreased for all patients, and this phenomenon was not as obvious as that in the other groups in group A (*P* < 0.05).

### 3.6. Wound Area and Healing Time

**Figure 8:**
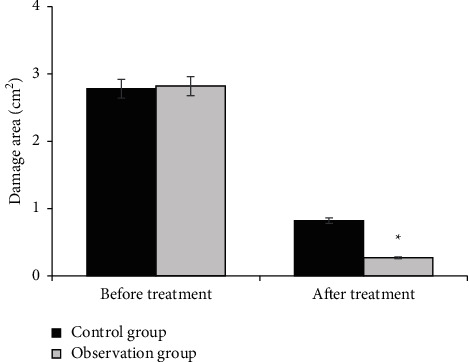
Comparison of the area of hand pressure injury between the two groups before and after treatment (^*∗*^ denotes that compared with control patients, *P* < 0.05).

**Figure 9:**
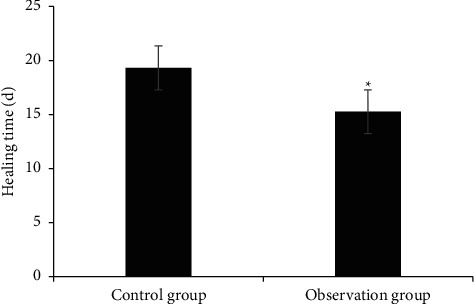
Comparison of healing time of hand pressure injury between the two groups (^*∗*^denotes that compared with control patients, *P* < 0.05).

Figure 8 shows the comparison of the area of hand pressure injury between the two groups before and after treatment, and Figure 9 shows the comparison of the healing time of hand pressure injury between the two groups. The area of hand pressure injury before treatment was 2.78 cm^2^ in the control group and 2.82 cm^2^ in the observation group. After treatment, the area of hand pressure injury in the control group was 0.82 cm^2^ and that in the observation group was 0.27 cm^2^. The area of hand pressure injury was dramatically reduced in the two groups, and the reduction was greater in the observation group (*P* < 0.05). The healing time of hand pressure injury in the control group was 19.32 days and that in the observation group was 15.27 days. The healing time of hand pressure injury in the observation group was dramatically shorter than that in the control group (*P* < 0.05).

## 4. Discussion

A pressure injury is an injury caused by various pressures on the body. Pressure, shear force, and friction force all cause pressure injury to patients [16]. Prolonged pressure on the body is not conducive to blood circulation. Ischemia and hypoxia cause nutritional deficiency, difficult wound recovery, tissue damage, and kidney necrosis [17]. Western medicine mainly promotes wound recovery by debridement and surgical treatment, while traditional Chinese medicine promotes wound healing by applying traditional Chinese medicine. The application of traditional Chinese medicine has low cost, simple operation, few side effects, and little damage to patients. It has good effects and high clinical application value. Among them, Dragon's Blood is a commonly used traditional Chinese medicine for the treatment of pressure injury, which can promote blood circulation, disperse blood stasis, relieve pain, stop bleeding, generate muscles, and collect sores.

The wound healing process consists of four stages, namely, hemostasis, inflammation, proliferation, and remodeling, and many wound dressings and techniques have been developed to enhance the body's ability to close wounds and restore function to damaged tissues. The growing threat of bacterial infections and chronic wound healing has triggered an urgent need for novel antimicrobial wound dressings. Shi et al. [18] developed a wound dressing for the treatment of infected wounds that can reduce the inflammatory phase (by using gentamicin sulfate (GS)) and enhance the granulation phase (by adding platelet-rich plasma (PRP)), reduce the number of bacteria, inhibit proinflammatory factors, and enhance anti-inflammatory factors. This study demonstrated the great potential of biocompatible wound dressings with the dual release of GS and PRP for the treatment of chronic and infected wounds. In this study, the therapeutic effects of sterile gauze treatment and Dragon's Blood treatment on pressure hand injury were explored. The results showed that the number of patients in the observation group who completely disappeared from local symptoms were dramatically higher than that in the control group, and the number of patients without clinical effects was dramatically lower than that in the control group (*P* < 0.05). The effective rate of the control group was 80% and that of the observation group was 93.33%. The effective rate of the observation group was dramatically higher than that of the control group (*P* < 0.05). It was concluded that Dragon's Blood was dramatically better than sterile gauze in the treatment of hand pressure injury and can promote the recovery of patients. After treatment, the number of patients with good granulation tissue in the observation group was dramatically greater than that in the control group (*P* < 0.05). After treatment, the number of patients with a small amount of exudation at the injury site in the observation group was dramatically greater than that in the control group (*P* < 0.05). After treatment, the number of patients without edema in the observation group was dramatically higher than that in the control group (*P* < 0.05). This shows that Dragon's Blood treatment can promote the generation of fibrocytes in pressure hand injury and promote wound recovery. Clearly, Dragon's Blood can promote wound recovery in patients with hand pressure injury, relieve edema, and reduce the amount of wound exudation.

MMPs and TIMPs are involved in the degradation of various proteins in the extracellular matrix (ECM) and play important roles in angiogenesis, embryogenesis, morphogenesis, and tissue remodeling in wound repair. The dynamic balance of MMPs and TIMPs has an important impact on wound repair [19]. The ECM plays an important role in cell differentiation, proliferation, morphological changes, and phenotypic expression. TGF-*β*1 is closely related to the growth and reproduction of fibrocytes, has a regulatory effect on the growth of vascular endothelial cells, can affect the formation of scars, and has an important role in wound recovery. VEGF can promote the division and proliferation of vascular endothelial cells and provide a material basis for the formation of blood vessels. TGF-*β*1 and VEGF are closely related to the generation of blood vessels. Dragon's Blood can promote the formation of fibroblasts and improve the epidermal repair ability near wounds by promoting the expression of VEGF. Ding et al. [20] explored the regulatory mechanism between TGF-*β*1 and VEGF and the generation of blood vessels and found that the overexpression of specific protein 1 (SP1) or TGF-*β*1 increased the expression level and secretion of VEGF, and VEGF could promote the division and proliferation of vascular endothelial cells, providing a material basis for the formation of blood vessels. The results of this study showed that there were no statistically significant differences in MMP-3, T1MP-1, VEGF, and TGF-B1 levels between the two groups before treatment (*P* > 0.05). After treatment, the levels of MMP-3, VEGF, and TGF-B1 were dramatically increased, especially in the observation group (*P* < 0.05). The level of T1MP-1 decreased, especially in the observation group (*P* < 0.05). After treatment, the area of pressure hand injury in the control group was 0.82 cm^2^ and that in the observation group was 0.27 cm^2^. The area of pressure hand injury in both groups was reduced, and the reduction in the observation group was greater (*P* < 0.05). The healing time of pressure hand injury in the control group was 19.32 days and that in the observation group was 15.27 days. The healing time of pressure hand injury in the observation group was dramatically shorter (*P* < 0.05). Dragon's Blood had a good effect on the treatment of hand pressure wounds, which is worthy of popularization and application.

## 5. Conclusion

In this study, the therapeutic effects of Dragon's Blood and sterile gauze in the treatment of pressure hand injury were compared, and the research significance and contribution were also analyzed. It was found that Dragon's Blood had a good effect on the treatment of pressure hand injury, and the effective rate was dramatically higher than that of sterile gauze dressing. In addition, the wound recovery was good, the amount of exudate in the wound was dramatically reduced, the degree of edema was alleviated, the area of the wound was dramatically reduced, and the wound recovered quickly. Dragon's blood has positive clinical significance and can provide treatment options for wound recovery in patients with pressure injury. The limitation of this study is that it only compared the different effects of Dragon's Blood and sterile gauze, without comparing the therapeutic effects of other wound treatment drugs. In the future, it is necessary to compare more kinds of wound healing drugs to explore the best pressure injury treatment drugs.

## Figures and Tables

**Table 1:** Comparison of general data between the two groups.

	Systolic pressure (mmHg)	Diastolic pressure (mmHg)	Blood sugar (mmol/L)
Control group	123.24 ± 12.79	82.33 ± 7.79	4.33 ± 1.69
Observation group	124.66 ± 12.38	81.46 ± 7.56	4.54 ± 1.65

## Data Availability

The data used to support the findings of this study are included within the article.

## References

[1] Sozbilen M. C., Dastan A. E., Gunay H., Kucuk L. (2021). One-year prospective analysis of hand and forearm injuries in children. *Journal of Pediatric Orthopaedics B*.

[2] Luria S., Talmud D., Volk I., Liebergall M., Calderon-Margalit R. (2019 Jan 9). The epidemiology of wrist and hand injury in two hospitals in Jerusalem: substantial differences between population subgroups. *Israel Journal of Health Policy Research*.

[3] Zhang J., Ren J., Liu Y., Huang D., Lu L. (2020 Sep 1). Resveratrol regulates the recovery of rat sciatic nerve crush injury by promoting the autophagy of Schwann cells. *Life Sciences*.

[4] Lee J. K., Hwang D., Han S. H., Lee Y. (2022 Apr). Complete occlusion of radial and ulnar arteries following hand crush injury with multiple carpometacarpal joint fracture-dislocations. *Journal of Hand Surgery*.

[5] Güntürk ÖB., Kaplan İ, Yıldırım T., Gürbüz Y., Ademoğlu Y., Ada S. (2021 Dec). Reconstruction of mutilating hand injuries by microsurgical free tissue transfers from the foot. *Injury*.

[6] Shrestha S., Tamrakar S., Banskota A. K. (2019 Nov 13). Outline of hand and wrist injuries presenting to an emergency of a tertiary care centre in Nepal. *Journal of Nepal Health Research Council*.

[7] Kerschhagl M., Larcher L., Mattiassich G., Prantl L. (2019). Replantation of a circumferentially degloved thumb in an occupational crush injury - a case report and review of the literature. *Clinical Hemorheology and Microcirculation*.

[8] Messana F., Faccio D., Rizzato S. (2021). Combining traditional and microsurgical reconstruction after a complex hand trauma with multiple tissue defects. A case report. *Annali Italiani di Chirurgia*.

[9] Zhou H., Yu T. (2022). Effect of comprehensive rehabilitation training program in orthopedic nursing of patients with residual limb injury caused by crush. *Journal of Healthcare Engineering*.

[10] Dizin F., Saab M., Mézel A., Guerre E., Chantelot C. (2022). Epidemiology of pediatric hand surgery emergencies. Retrospective study of 245 patients seen over 10 months in two referral centers. *Orthopaedics and Traumatology: Surgery & Research*.

[11] Sharma A., Sankhe M. (2022). A proposed classification for sugarcane crusher injuries of the hand and its correlation with patient rated outcome scores at 6 months. *Journal of Hand Surgery*.

[12] Yildirimer L., Brewster C. T., Aziz H., Unluer Z., Jemec B., De Leo A. (2019). Experience of nail bed injuries at a tertiary hand trauma unit: a 12-month review and cost analysis. *Journal of Hand Surgery*.

[13] Feldman G., Hitti S., Rozen N., Rubin G. (2020). Molten metal high pressure injection injury of the hand. *Hand Surgery and Rehabilitation*.

[14] Munoz N., Posthauer M. E., Cereda E., Schols J. M. G. A., Haesler E. (2020). The role of nutrition for pressure injury prevention and healing: the 2019 international clinical practice guideline recommendations. *Advances in Skin & Wound Care*.

[15] Tschannen D., Anderson C. (2020). The pressure injury predictive model: a framework for hospital-acquired pressure injuries. *Journal of Clinical Nursing*.

[16] Munoz N., Posthauer M. E. (2021). Nutrition strategies for pressure injury management: implementing the 2019 international clinical practice guideline. *Nutrition in Clinical Practice*.

[17] Pona A., Cline A., Kolli S. S., Taylor S. L., Feldman S. R. (2019 Mar). Review of future insights of Dragon’s Blood in dermatology. *Dermatologic Therapy*.

[18] Shi L., Lin F., Zhou M. (2021). Preparation of biocompatible wound dressings with dual release of antibiotic and platelet-rich plasma for enhancing infected wound healing. *Journal of Biomaterials Applications*.

[19] Bassiouni W., Ali M. A. M., Schulz R. (2021). Multifunctional intracellular matrix metalloproteinases: implications in disease. *FEBS Journal*.

[20] Ding A., Bian Y. Y., Zhang Z. H. (2020). SP1/TGF-*β*1/SMAD2 pathway is involved in angiogenesis during osteogenesis. *Molecular Medicine Reports*.

